# Novel Potential Diagnostic Serum Biomarkers of Metabolomics in Osteoarticular Tuberculosis Patients: A Preliminary Study

**DOI:** 10.3389/fcimb.2022.827528

**Published:** 2022-03-25

**Authors:** Ximeng Chen, Jingyun Ye, Hong Lei, Chengbin Wang

**Affiliations:** ^1^ Medical School of Chinese People’s Liberation Army (PLA), Beijing, China; ^2^ Department of Clinical Laboratory Medicine, The First Medical Center, Chinese People’s Liberation Army (PLA) General Hospital, Beijing, China; ^3^ Department of Clinical Laboratory Medicine, The Eighth Medical Center, Chinese People’s Liberation Army (PLA) General Hospital, Beijing, China

**Keywords:** osteoarticular tuberculosis, metabolomics, biomarker (BM), lipid metabolism, tuberculosis

## Abstract

Osteoarticular tuberculosis is one of the extrapulmonary tuberculosis, which is mainly caused by direct infection of *Mycobacterium tuberculosis* or secondary infection of tuberculosis in other parts. Due to the low specificity of the current detection method, it is leading to a high misdiagnosis rate and subsequently affecting the follow-up treatment and prognosis. Metabolomics is mainly used to study the changes of the body’s metabolites in different states, so it can serve as an important means in the discovery of disease-related metabolic biomarkers and the corresponding mechanism research. Liquid chromatography tandem mass spectrometry (LC-MS/MS) was used to detect and analyze metabolites in the serum with osteoarticular tuberculosis patients, disease controls, and healthy controls to find novel metabolic biomarkers that could be used in the diagnosis of osteoarticular tuberculosis. Our results showed that 68 differential metabolites (*p*<0.05, fold change>1.0) were obtained in osteoarticular tuberculosis serum after statistical analysis. Then, through the evaluation of diagnostic efficacy, PC[o-16:1(9Z)/18:0], PC[20:4(8Z,11Z,14Z,17Z)/18:0], PC[18:0/22:5(4Z,7Z,10Z,13Z,16Z)], SM(d18:1/20:0), and SM[d18:1/18:1(11Z)] were found as potential biomarkers with high diagnostic efficacy. Using bioinformatics analysis, we further found that these metabolites share many lipid metabolic signaling pathways, such as choline metabolism, sphingolipid signaling, retrograde endocannabinoid signaling, and sphingolipid and glycerophospholipid metabolism; these results suggest that lipid metabolism plays an important role in the pathological process of tuberculosis. This study can provide certain reference value for the study of metabolic biomarkers of osteoarticular tuberculosis and the mechanism of lipid metabolism in osteoarticular tuberculosis and even other tuberculosis diseases.

## Introduction

Tuberculosis is a chronic infectious disease that is caused by *Mycobacterium tuberculosis (M.tb)* and takes the respiratory tract as the main route of transmission (Walzl et al., 2018). According to the Global Tuberculosis Report by the World Health Organization in 2021, 9.9 million new cases and 1.5 million deaths were caused by tuberculosis globally in 2020 (World Health Organization, 2021). Generally, tuberculosis is divided into two types: pulmonary tuberculosis (PTB) and extrapulmonary tuberculosis (EPTB). For the diagnosis of tuberculosis, clinical laboratory tests play an important role, although with the long culture time and the low positive rate, the culture of *Mycobacterium tuberculosis* is still the gold standard (Darton et al., 2017); the other common tests such as smear microscopy, purified protein derivative (PPD) test, interferon-gamma (IFN-γ) release assay (IGRA), *M.tb* nucleic acid test, and Xpert/MTB system are also widely used (Fan et al., 2012; Norbis et al., 2014; Fan et al., 2018).

Osteoarticular tuberculosis is a kind of extrapulmonary tuberculosis whose *M.tb* directly infects the bone and joint tissue or spreads to the bone and joint tissue from other parts, which accounts for about 3%–5% of the total incidence of EPTB (Norbis et al., 2014). The most common site for osteoarticular tuberculosis is spine, especially the thoracic and lumbar spine, and the pathological changes are mostly bone destruction and tuberculous granulation tissue formation. At the same time, tuberculous abscesses that do not show related inflammation are easily formed beside the bone, and in severe cases, sinus tracts may even be formed (Johansen et al., 2015). The early clinical manifestations of osteoarticular tuberculosis are atypical, and the specificity of laboratory tests and imaging examinations is low, resulting in a high rate of missed diagnosis for osteoarticular tuberculosis. Most patients are misdiagnosed during the advanced stage that affected the treatment and prognosis. Hence, there is an urgent need for accurate diagnosis methods of osteoarticular tuberculosis (Yi et al., 2018; Vinhaes et al., 2019).

Metabolomics is mainly used to study the changes of the body’s metabolites in different states (Zhang et al., 2018); the main methods of metabolomics contained nuclear magnetic resonance (NMR) and mass spectrometry (MS) (Zhou and Yin, 2019). Numerous metabolites have been successfully discovered as biomarkers for the diagnosis of various diseases. What is more, the metabolites which are searched by metabolomics can be elucidating the pathological or functional mechanisms by bioinformatics analysis (Wheelock et al., 2013). In the aspect of tuberculosis, there were many metabolomics studies of PTB, including secondary pulmonary tuberculosis (Luies et al., 2017), active pulmonary tuberculosis (Cho et al., 2020), and drug-resistant pulmonary tuberculosis (Tuyiringire et al., 2018; Chen et al., 2020); however, for osteoarticular tuberculosis, because of the low incidence of this disease, the related metabolomics studies are rare.

According to this situation, we used LC-MS/MS to detect serum metabolites in osteoarticular tuberculosis patients (TB Group), osteoarthritis patients (DC Group, including rheumatoid arthritis and ankylosing spondylitis), and healthy controls (HC Group) (Dudka et al., 2021), aiming to find potential markers for early and accurate diagnosis of osteoarticular tuberculosis. We further compared and analyzed serum metabolites of the above three groups and selected differential metabolites by the bioinformatics method to analyze the corresponding pathogenesis (Zhang et al., 2019).

## Materials and Methods

### Study Cohort

From November 2018 to November 2019, 30 serum samples of diagnosed osteoarticular tuberculosis patients (TB group), 30 serum samples of disease control containing rheumatoid arthritis patients and ankylosing spondylitis patients (DC group), and 30 serum samples of healthy control (HC group) were collected from the 1st and the 8th Medical Center of Chinese PLA General Hospital. The diagnosis of the TB group was based on the following criteria: (a) positive nucleic acid test of *M.tb*; (b) medical image (X-ray, CT scan, etc.) findings showed specific features of TB infection; (c) positive pathology diagnosis of TB in bone or joint specimens; and (d) effective response to antituberculosis treatments. The diagnosis of the DC group was based on the following criteria: (a) positive inflammatory protein test; (b) specific clinical manifestations (joint stiffness in the morning, bending change, etc.); (c) medical image (X-ray, CT scan, etc.) findings showed specific features; and (d) effective response to hormone treatments (glucocorticoid, etc.). The HC group included adults without any disease clinical diagnosis, and all the tests were negative or normal. Patients with any diagnosis of cancer, metabolic disease, autoimmunity disease, immunodeficiency disease, and other pathogen infections were excluded from this study. Patients who have other organs tuberculosis (e.g., pulmonary tuberculosis or other extrapulmonary tuberculosis) were excluded as well.

This research was carried out in strict accordance with the declaration of Helsinki and approved by the Ethics Committee of Chinese PLA General Hospital. All participants signed an informed consent and gave their permission to use their blood samples for this study.

For each patient, 5 ml peripheral blood was drawn under a vacuum vessel containing separation gel in the morning before any treatments. After blood coagulation and 2,370g centrifugation, the serum was divided into several EP tubes and stored in a -80°C refrigerator for subsequent metabolomics analysis.

### Metabolomics Analysis

This study applied untargeted metabolomics, which included reversed-phase chromatography positive ion detection, reversed-phase chromatography anion detection, and hydrophilic chromatography positive ion detection. The types of metabolites detected by these three modes are positive ion lipid, negative ion lipid (e.g., fatty acid), and small polar molecules (e.g., amino acid), respectively. The analysis contained three parts: serum pretreatment and separation, mass spectrometry detection, and data processing.

#### Serum Pretreatment and Separation

Serum samples were thawed at 4°C, 300 µl methanol and 1,000 µl methyl tert-butyl ether (reversed-phase chromatography ion) or 150 µl acetonitrile (hydrophilic chromatography ion) was added to get a mixture in a microcentrifuge tube. Then, the mixture was centrifuged at 4°C temperature, 12,000 rpm for 10 min, and 100 μl supernatant was taken for analysis. As for reversed-phase chromatography ion mode, 400 µl was first taken from the mixture to dry, and then 100 μl methanol was added to dissolve after centrifugation.

For reversed-phase chromatography ion separation, mobile phase A was acetonitrile/water (60/40) and mobile phase B was isopropanol/acetonitrile (90/10); both A and B contained 0.1% formic acid and 10 mmol/l ammonium acetate. The column was an HSS T3 column (2.1 × 100 mm, 1.8 µm) operated at 45°C. The flow rate was 300 µl/min, and the injection volume was 1 µl. For hydrophilic chromatography ion separation, mobile phase A was acetonitrile and mobile phase B was water; both A and B contained 0.1% formic acid and 10 mmol/l ammonium acetate. The column was a BEH Amide column (2.1 × 100 mm, 1.7 µm) operated at 40°C. The flow rate was 300 µl/min, and the injection volume was 1 µl.

#### Mass Spectrometry Detection

A Thermo Scientific™ Q Exactive™ Hybrid Quadrupole-Orbitrap Mass Spectrometer equipped with a HESI-II probe was employed. The positive and negative HESI-II spray voltages were 3.7 and 3.5 kV, respectively, the heated capillary temperature was 320°C, the sheath gas pressure was 30 psi, the auxiliary gas setting was 10 psi, and the heated vaporizer temperature was 300°C. Both the sheath gas and the auxiliary gas were nitrogen. The collision gas was also nitrogen at a pressure of 1.5 mTorr. The parameters of the full mass scan were as follows: a resolution of 70,000, an auto gain control target under 1 × 10^6^, a maximum isolation time of 50 ms, and an m/z range 50–1500. The LC-MS system was controlled using Xcalibur 2.2 SP1.48 software (Thermo Fisher Scientific, Waltham, MA, USA), and data were collected and processed with the same software.

#### Data Processing

All data obtained from the four assays in the two systems in both positive and negative ion modes were processed using Progenesis QI data analysis software (Nonlinear Dynamics, Newcastle, UK) for imputing raw data, peak alignment, picking, and normalization to produce peak intensities for retention time (tR) and m/z data pairs. The ranges of automatic peak picking for C18 were between 1 and 16 min and between 1 and 12 min, respectively. Then, the adduct ions of each feature (*m/z*, tR) were deconvoluted, and these features were identified in the Human Metabolome Database (HMDB, http://www.hmdb.ca/) and LIPID MAPS (http://www.lipidmaps.org/).

To monitor the system’s stability and performance and the reproducibility of the sample, quality control (QC) samples were prepared by pooling equal volumes of each serum sample. The pretreatment of serum QC samples was in accord with real samples. For repeatable metabolic analyses, three features of the analytical system must be stable: (1) retention time, (2) signal intensity, and (3) mass accuracy. In this study, three QCs were continuously injected at the beginning of the run. QC samples are then injected at regular intervals of six or eight samples throughout the analytical run-in order to provide data from which repeatability can be assessed.

The features were selected based on their coefficients of variation (CVs) with QC samples; features with CVs over 15% were eliminated.

### Statistical Analysis

The chi-square test was used for the analysis of characteristics of the study participants, and the Kruskal–Wallis H test was used to determine the differences between groups. The data of metabolomics were normalized using Progenesis QI data analysis software (Nonlinear Dynamics, Newcastle, UK). SIMCA 14.1 software was used to analyze the metabolites using the orthogonal partial least squares (OPLS) model. MetaboAnalyst (http://www.metaboanalyst.ca/) was used to analyze the related pathways of specific metabolites. R^2^X (the interpretability of the model for the categorical variable X) was obtained after cross-validation; R^2^Y (the interpretability of the model for the categorical variable Y) and Q^2^ (predictability of the model) were obtained after cross-validation to judge the validity of the model. The Variable Importance in the Projection (VIP) value and the *p* value of t-test were used for evaluating the difference metabolites between groups. Final results were shown with scatter plots, trend charts, and the receiver operator characteristic curve (ROC curve), and analyses of the AUC, sensitivity, and specificity of each different metabolites were made by GraphPad Prism 6.0 software. The establishment of diagnostic models which contained metabolic biomarkers was made by MedCalc software, including several different metabolites combination and its statistical analysis; meanwhile, logistic regression and ROC curve analysis were used for the establishment of diagnostic models.

## Results

### Basic Data Preprocessing

There was no statistical difference in the age and gender between TB, DC, and HC groups (*p*>0.05). The positive rates of clinical laboratory tests and medical imaging features are shown in **Table 1**. After LC-MS/MS analysis and peak alignment, picking, and normalization of raw data, metabolites were obtained, while the QC results of three patterns showed good reproducibility, which indicated that the results were credible (**Supplementary Files**).

**Table 1:** Clinical information of the study cohort.

	TB Group (n = 30)	DC Group (n = 30)	HC Group (n = 30)	*p* value
Age (median, IQR)	46 (33–61)	53.5 (31.5–63.5)	48.5 (41–55)	>0.05
Gender (male/female)	16/14	14/16	17/13	>0.05
Xpert test positive no. (%)	17 (56.67%)	/	/	/
TB nucleic acid test positive no. (%)	13 (43.33%)	/	/	/
TB antibody test positive no. (%)	9 (30.0%)	/	/	/
IGRA positive no. (%)	25 (83.3%)	/	/	/
Cultivate positive no. (%)	2 (6.67%)	/	/	/
Imaging features positive no. (%)	24 (80.00%)	/	/	/
Pathology positive no. (%)	17 (56.67%)	/	/	/

### Metabolite Profile

**Figure 1:**
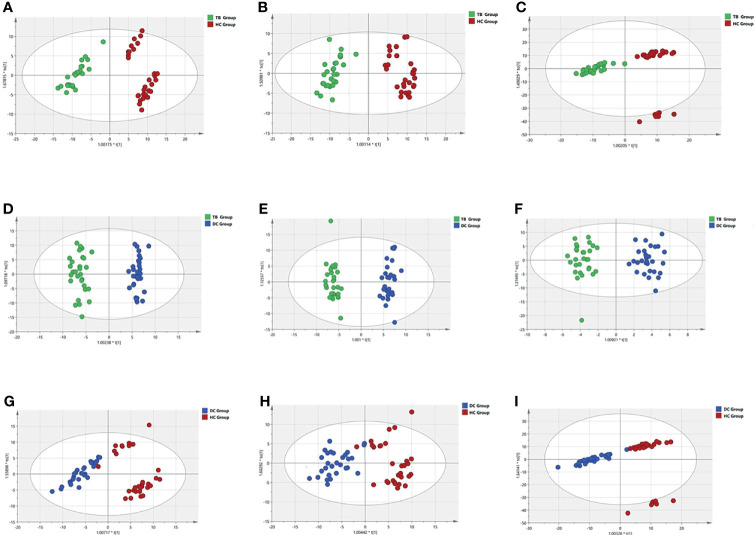
OPLS-DA models of three patterns in three groups. **(A, D, G)** reversed-phase chromatography positive ion; **(B, E, H)** reversed-phase chromatography anion; **(C, F, I)** hydrophilic chromatography positive ion.

The OPLS-DA models of three patterns showed that the metabolites in three groups were clearly separated (**Figure 1**), which indicated that significant serum metabolites change in osteoarticular tuberculosis patients. Variable Importance in the Projection (VIP) is a factor that means to extend a variable contribute in the projection, and the *p* <0.05 of statistical tests between groups is also important to explain the differences. The R^2^Y and Q^2^ of each OPLS-DA model are shown in **Table 2**; as TB compared to HC, the R^2^Y were 0.936, 0.945, and 0.906, respectively, and the Q^2^ were 0.870, 0.898, and 0.877, respectively; as TB compared to DC, the R^2^Y were 0.972, 0.976, and 0.941 respectively, and the Q^2^ were 0.956, 0.965, and 0.690, respectively; and as DC compared to HC, the R^2^Y were 0.824, 0.817, and 0.832, respectively, and the Q^2^ were 0.620, 0.646, and 0.789, respectively.

**Table 2:** OPLS-DA models parameters.

	Reversed-phase chromatography Positive ion		Reversed-phase chromatography Anion		Hydrophilic chromatography Positive ion	
	R^2^Y	Q^2^	R^2^Y	Q^2^	R^2^Y	Q^2^
TB vs. HC	0.936	0.870	0.945	0.898	0.906	0.877
TB vs. DC	0.972	0.956	0.976	0.965	0.941	0.690
DC vs. HC	0.824	0.620	0.817	0.646	0.832	0.789

**Figure 2:**
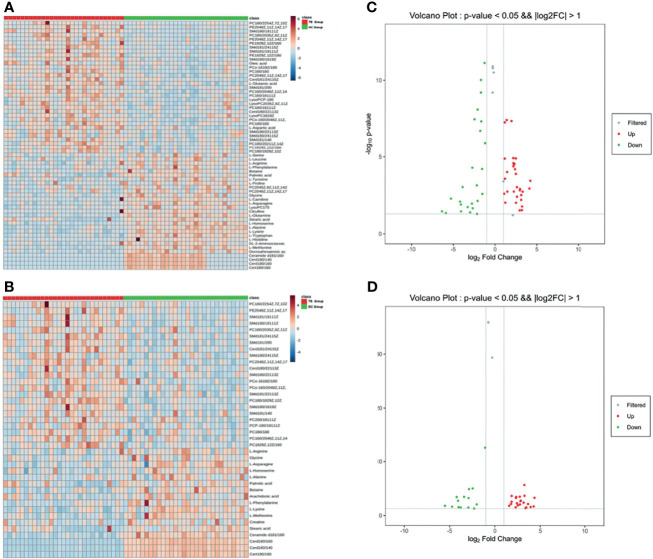
Heat maps and volcano maps of metabolites. **(A)** Heat map of TB group vs. HC group; **(B)** volcano map of TB group vs. HC group **(C)** heat map of TB group vs. DC group; **(D)** volcano map of TB group vs. DC group.

**Figure 3:**
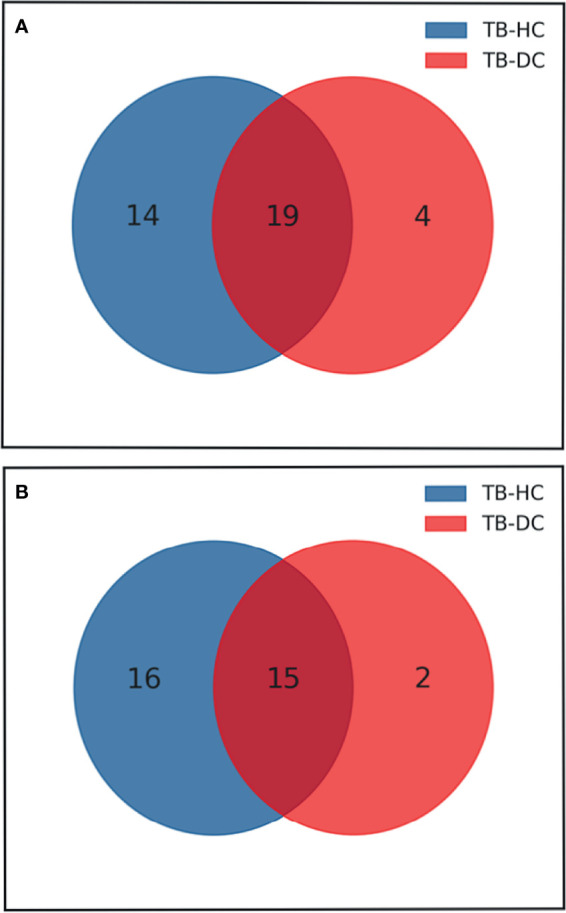
Venn diagram of differential metabolites between TB group vs HC group and TB group vs DC group. **(A)** Up-regulated metabolites; **(B)** Down-regulated metabolites

According to the results of the LC-MS/MS analysis, 62 and 40 metabolites were obtained among TB vs. HC and TB vs. DC, respectively; the heat maps and volcano maps are shown in **Figure 2**. After screening by a difference standard, 68 differential metabolites were obtained among TB vs. HC and TB vs. DC, including 37 upregulated metabolites and 31 downregulated metabolites. Further, based on the Venn diagram of these two comparisons (**Figure 3**), 19 upregulated common metabolites and 15 downregulated common metabolites were found (**Table 3**). The upregulated metabolites were phosphatidylcholine (PC), phosphatidylethanolamine (PE), ceramide (Cer), sphingomyelin (SM), etc. The downregulated metabolites were amino acid, ceramide (Cer), and fatty acid. At the same time, we conducted basic information retrieval and statistical analysis of 34 differential metabolites.

**Table 3:** Common metabolites between TB vs. HC and TB vs. DC.

Metabolite	HMDB ID	Log_2_ fold change		Type
		TB vs. HC	TB vs. DC	
PC(o-16:1(9Z)/18:0)	HMDB13412	2.172	2.839	Up
PC(20:4(8Z,11Z,14Z,17Z)/18:0)	HMDB08464	2.192	2.745	Up
PC(16:0/20:4(8Z,11Z,14Z,17Z))	HMDB07983	2.383	4.281	Up
PC(18:0/18:2(9Z,12Z))	HMDB08039	4.213	3.249	Up
PC(18:2(9Z,12Z)/16:0)	HMDB08133	4.107	4.362	Up
PC(18:0/20:3(5Z,8Z,11Z))	HMDB08046	1.154	2.288	Up
PC(18:0/22:5(4Z,7Z,10Z,13Z,16Z))	HMDB08055	0.988	1.570	Up
PC(o-16:0/20:4(8Z,11Z,14Z,17Z))	HMDB13407	2.970	2.854	Up
SM(d18:1/20:0)	HMDB12102	2.317	2.354	Up
SM(d18:1/24:1(15Z))	HMDB12107	1.383	2.318	Up
SM(d18:0/16:1(9Z))	HMDB13464	1.953	3.265	Up
SM(d18:0/18:1(11Z))	HMDB12088	1.151	1.843	Up
SM(d18:0/24:1(15Z))	HMDB12095	3.213	2.527	Up
SM(d18:0/22:1(13Z))	HMDB12092	3.133	2.822	Up
SM(d18:1/18:1(11Z))	HMDB12100	1.436	1.840	Up
SM(d18:1/14:0)	HMDB12097	3.234	3.371	Up
Cer(d18:1/24:1(15Z))	HMDB04953	2.241	2.428	Up
Cer(d18:0/22:1(13Z))	HMDB11766	2.618	2.759	Up
PE(20:4(8Z,11Z,14Z,17Z)/18:0)	HMDB09420	1.174	1.773	Up
Glycine	HMDB00123	-2.730	-4.723	Down
L-Arginine	HMDB00517	-5.286	-5.514	Down
L-Alanine	HMDB00161	-1.708	-4.011	Down
L-Phenylalanine	HMDB00159	-4.203	-2.912	Down
L-Asparagine	HMDB00168	-2.475	-4.218	Down
L-Homoserine	HMDB00719	-1.781	-4.070	Down
L-Methionine	HMDB00696	-1.273	-2.458	Down
L-Lysine	HMDB00182	-1.706	-2.862	Down
Betaine	HMDB00043	-4.098	-3.404	Down
Cer(d18:0/14:0)	HMDB11759	-0.292	-0.719	Down
Cer(d18:0/16:0)	HMDB11760	-0.291	-0.752	Down
Cer(t18:0/16:0)	HMDB10697	-0.178	-0.305	Down
Cer(d18:1/16:0)	HMDB04949	-0.307	-1.077	Down
Palmitic acid	HMDB00220	-4.082	-3.782	Down
Stearic acid	HMDB00827	-2.099	-2.011	Down

### KEGG Enrichment Analysis

**Figure 4:**
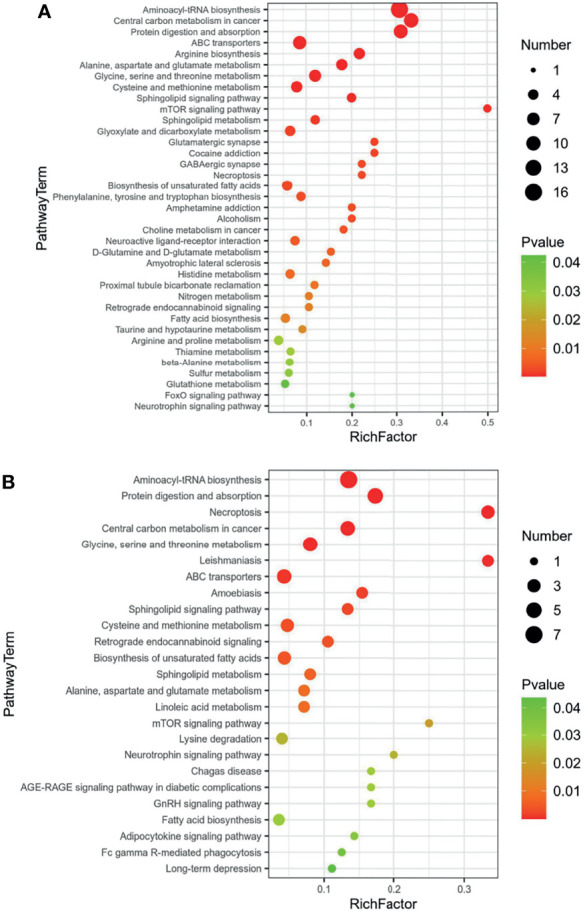
KEGG enrichment of common regulated metabolites **(A)** Up-regulated metabolites; **(B)** down-regulated metabolites.

For the differential metabolites screened out in the previous analysis, we conducted a bioinformatics analysis on the differential metabolites. We imported these metabolites one by one into the KEGG database for signal pathway analysis and performed further enrichment and statistical analysis of these results. After KEGG enrichment analysis, the main metabolic pathways in these differential metabolites are necroptosis, choline metabolism, sphingolipid signaling, retrograde endocannabinoid signaling, sphingolipid metabolism, and glycerophospholipid metabolism (**Figure 4**). According to the results of bioinformatics, it can be seen that the main signal pathways are concentrated in cell metabolism and lipid metabolism, which is in good agreement with the currently known pathogenic mechanisms of *Mycobacterium tuberculosis*.

### Metabolites Diagnostic Efficiency Evaluation

We performed ROC curve analysis, 95% CI value calculation, sensitivity and specificity analyses on the differential metabolites in the TB group according to the aforementioned results to evaluate the diagnostic efficacy of each differential metabolite (**Table 4**). Based on clinical practicability and feasibility, for the diagnostic efficacy evaluation of each differential metabolite, we focus on the differential metabolites that specifically increase in osteoarticular tuberculosis. Generally, an AUC value greater than 0.7 indicates better diagnostic performance; according to this principle, SM(d18:0/24:1(15Z)) and SM(d18:1/14:0) were excluded from the evaluation. On the other hand, according to the comprehensive consideration of the AUC value, 95% CI, sensitivity, and specificity of each different metabolite, PC(o-16:1(9Z)/18:0), PC(20:4(8Z,11Z,14Z,17Z)/18:0), PC(18:0/20:3(5Z,8Z,11Z)), PC(18:0/22:5(4Z,7Z,10Z,13Z,16Z)), SM(d18:1/20:0), SM(d18:1/24:1(15Z)), SM(d18:0/16:1(9Z)), SM(d18:0/18:1(11Z)), and SM(d18:1/18:1(11Z)) were finally selected for further diagnostic efficacy evaluation and analysis.

**Table 4:** Diagnostic test information of metabolites between TB vs. HC and TB vs. DC.

Metabolites	AUC	95% CI	Sensitivity	Specificity
PC(o-16:1(9Z)/18:0)	0.8287	0.7235–0.9340	82.76%	76.67%
PC(20:4(8Z,11Z,14Z,17Z)/18:0)	0.7989	0.6858–0.9119	75.86%	73.33%
PC(16:0/20:4(8Z,11Z,14Z,17Z))	0.7770	0.6607–0.8933	68.97%	66.67%
PC(18:0/18:2(9Z,12Z))	0.7494	0.6251–0.8738	72.41%	66.67%
PC(18:2(9Z,12Z)/16:0)	0.7218	0.5924–0.8512	65.62%	66.67%
PC(18:0/20:3(5Z,8Z,11Z))	0.8529	0.7581–0.9477	79.31%	76.67%
PC(18:0/22:5(4Z,7Z,10Z,13Z,16Z))	0.8448	0.7423–0.9473	86.21%	76.67%
PC(o-16:0/20:4(8Z,11Z,14Z,17Z))	0.7195	0.5887–0.8504	65.52%	66.67%
SM(d18:1/20:0)	0.7989	0.6885–0.9092	75.86%	66.67%
SM(d18:1/24:1(15Z))	0.9057	0.8333–0.9782	82.76%	80.00%
SM(d18:0/16:1(9Z))	0.9107	0.8381–0.9833	85.71%	80.00%
SM(d18:0/18:1(11Z))	0.9506	0.9031–0.9980	82.76%	83.33%
SM(d18:0/24:1(15Z))	0.6529	0.5090–0.7968	62.07%	63.33%
SM(d18:0/22:1(13Z))	0.7189	0.5815–0.8484	72.41%	70.00%
SM(d18:1/18:1(11Z))	0.8138	0.7040–0.9235	72.41%	76.67%
SM(d18:1/14:0)	0.6621	0.5218–0.8023	82.76%	56.67%
Cer(d18:1/24:1(15Z))	0.7138	0.5811–0.8465	68.97%	66.67%
Cer(d18:0/22:1(13Z))	0.7195	0.5832–0.8559	72.41%	70.00%
PE(20:4(8Z,11Z,14Z,17Z)/18:0)	0.7690	0.6483–0.8896	72.41%	66.67%
Glycine	0.7954	0.6831–0.9077	72.41%	73.33%
L-Arginine	0.7402	0.6126–0.8679	68.97%	73.33%
L-Alanine	0.9172	0.8473–0.9872	86.21%	83.33%
L-Phenylalanine	0.6989	0.5658–0.8319	65.52%	63.33%
L-Asparagine	0.8989	0.8155–0.9822	82.76%	83.33%
L-Homoserine	0.9011	0.8226–0.9797	86.21%	86.67%
L-Methionine	0.9701	0.9309–1.0009	86.21%	93.93%
L-Lysine	0.9253	0.8620–0.9886	86.21%	86.67%
Betaine	0.6943	0.5595–0.8290	68.97%	63.33%
Cer(d18:0/14:0)	1	1	100%	100%
Cer(d18:0/16:0)	1	1	100%	100%
Cer(t18:0/16:0)	1	1	100%	100%
Cer(d18:1/16:0)	0.9782	0.9489–1.007	93.10%	96.67%
Palmitic acid	0.7115	0.5663–0.8567	68.97%	66.67%
Stearic acid	0.7678	0.6325–0.9032	72.41%	76.67%

**Figure 5:**
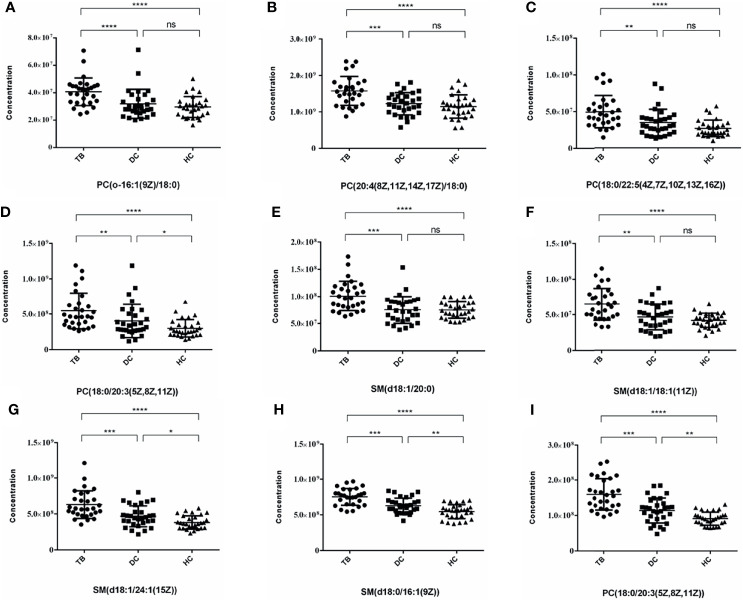
Scatter plots of 9 selected differential metabolites among TB, DC and HC Group. **(A)** PC(o-16:1(9Z)/18:0); **(B)** PC(20:4(8Z,11Z,14Z,17Z)/18:0); **(C)** PC(18:0/22:5(4Z,7Z,10Z,13Z,16Z)); **(D)** PC(18:0/20:3(5Z,8Z,11Z)) **(E)** SM(d18:1/20:0); **(F)** SM(d18:1/18:1(11Z)); **(G)** SM(d18:1/24:1(15Z)); **(H)** SM(d18:0/16:1(9Z)); **(I)** PC(18:0/20:3(5Z,8Z,11Z)) (*p < 0.05, **p < 0.01, ***p < 0.001, ****p < 0.0001, ns, no statistical difference).

After that, we drew scatter plots of the 9 selected differentially upregulated metabolites and performed a statistical analysis of their concentration in the three groups (**Figure 5**). As the scatter plots showed, PC(18:0/20:3(5Z,8Z,11Z)), SM(d18:1/24:1(15Z)), SM(d18:0/16:1(9Z)), and SM(d18:0/18:1(11Z)) also have a statistical difference between the DC group and HC group, which may disturb the diagnostic efficacy of osteoarticular tuberculosis. In contrast, the remaining five differential metabolites only have statistical difference between the TB group vs. DC group and TB group vs. HC group.

**Figure 6:**
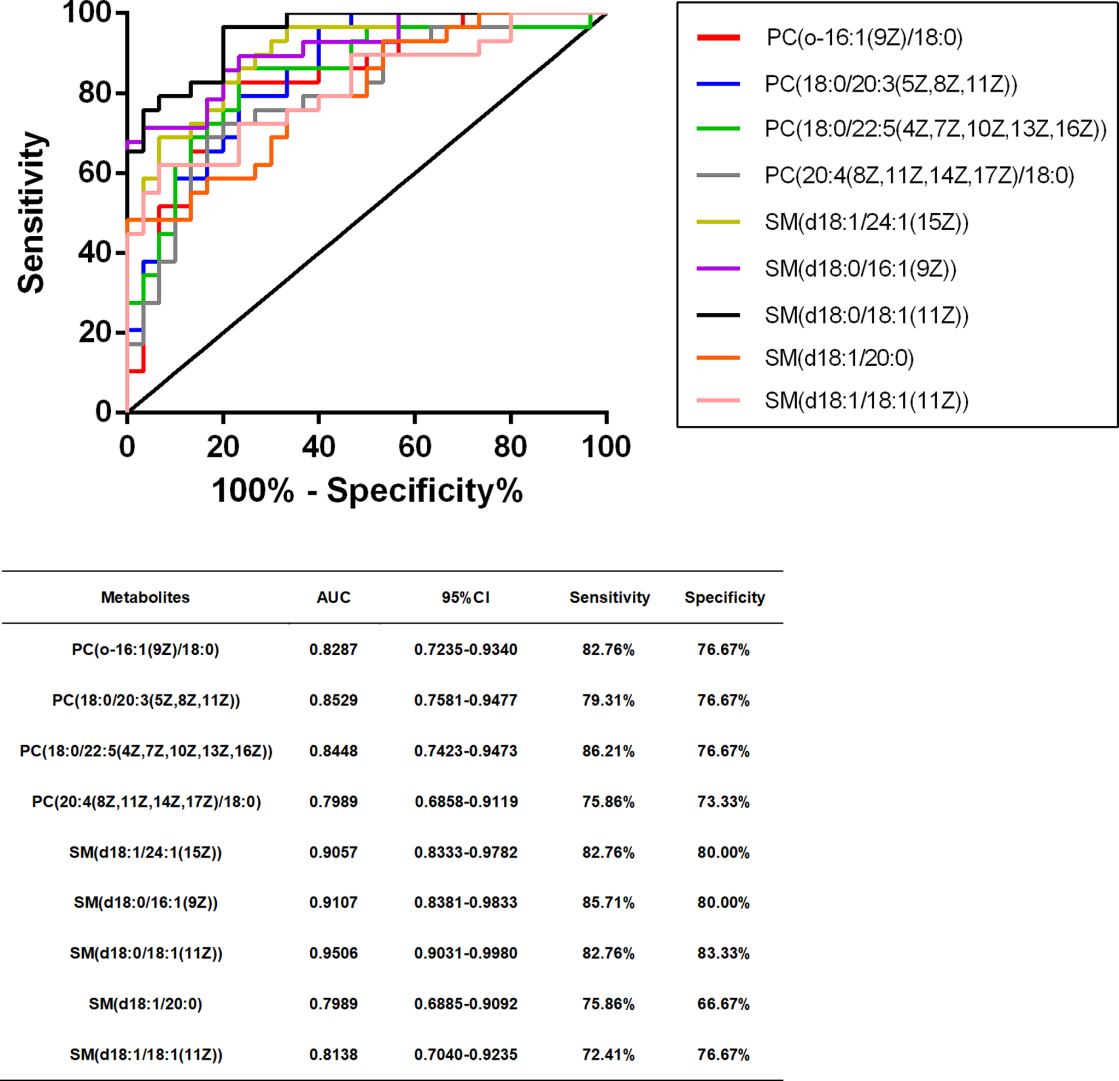
ROC curves of selected differential metabolites.

In summary, combining the AUC value of the ROC curve (**Figure 6**), 95% CI, sensitivity, and specificity, we finally considered that PC(o-16:1(9Z)/18:0), PC(20:4(8Z,11Z,14Z,17Z)/18:0), PC(18:0/22:5(4Z,7Z,10Z,13Z,16Z)), SM(d18:1/20:0), and SM(d18:1/18:1(11Z)) may be potentially relevant metabolic biomarkers for the diagnosis of osteoarticular tuberculosis.

### Establishment of Diagnostic Models Based on Five Potentially Metabolic Biomarkers

**Figure 7:**
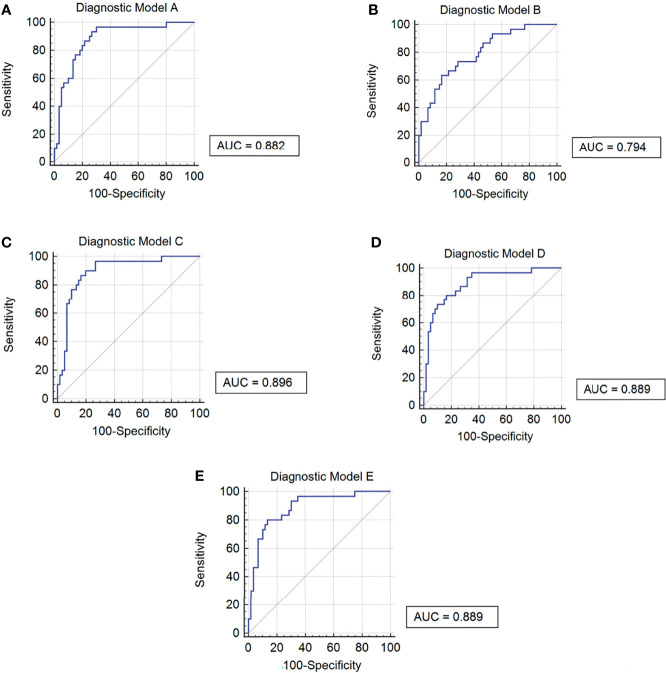
ROC curves of 5 diagnostic models. **(A)** Diagnostic Model A contained PC(o-16:1(9Z)/18:0), PC(20:4(8Z,11Z,14Z,17Z)/18:0) and PC(18:0/22:5(4Z,7Z,10Z,13Z,16Z)). **(B)** Diagnostic Model B contained SM(d18:1/20:0) and SM(d18:1/18:1(11Z)). **(C)** Diagnostic Model C contained PC(o-16:1(9Z)/18:0), PC(20:4(8Z,11Z,14Z,17Z)/18:0), PC(18:0/22:5(4Z,7Z,10Z,13Z,16Z)) and SM(d18:1/20:0). **(D)** Diagnostic Model D contained PC(o-16:1(9Z)/18:0), PC(20:4(8Z,11Z,14Z,17Z)/18:0) , PC(18:0/22:5(4Z,7Z,10Z,13Z,16Z)) and SM(d18:1/18:1(11Z)). **(E)** Diagnostic Model E contained PC(o-16:1(9Z)/18:0), PC(20:4(8Z,11Z,14Z,17Z)/18:0), PC(18:0/22:5(4Z,7Z,10Z,13Z,16Z)), SM(d18:1/20:0) and SM(d18:1/18:1(11Z)).

In order to improve the diagnostic efficiency of metabolites, diagnostic models are necessary to be considered. First of all, we combined two metabolites, PC and SM, respectively, to establish diagnostic models (Models A and B). After the combination of these metabolites, the AUC values of these two models were 0.8820 and 0.7940, respectively. However, the sensitivity of Model A and the specificity of Model B were more reasonable for the diagnosis, which were 96.67% and 83.33%, respectively. The data of Models A and B indicated that three PC biomarkers may be related to the sensitivity of models and two SM biomarkers may be related to the specificity of models. After that, for the improvement of the diagnostic efficiency, we added two SM biomarkers into PC combination respectively to establish new diagnostic models called Model C and Model D, which showed an ideal AUC value, 95% CI, sensitivity, and specificity (**Table 5**). What is more, the index of Model C is better than that of Model D comprehensively, indicating better diagnostic efficiency. Further, we also combined all the five biomarkers to establish Model E, although the AUC value, 95% CI, and sensitivity were similar to Model D, the specificity was improved to 86.67%. Also, the ROC curve of each diagnostic model was as shown in **Figure 7**. At last, based on the evaluation of these five diagnostic model indexes, we finally considered Model C as the most comprehensive diagnostic model in this study, consisting of PC(o-16:1(9Z)/18:0), PC(20:4(8Z,11Z,14Z,17Z)/18:0), PC(18:0/22:5(4Z,7Z,10Z,13Z,16Z)), and SM(d18:1/20:0).

**Table 5:** Diagnostic models based on five upregulated potential metabolic biomarkers.

Model	Components	AUC	95% CI	Sensitivity	Specificity
A	PC(o-16:1(9Z)/18:0)	0.8820	0.796–0.940	96.67%	70.00%
	PC(20:4(8Z,11Z,14Z,17Z)/18:0)				
	PC(18:0/22:5(4Z,7Z,10Z,13Z,16Z))				
B	SM(d18:1/20:0)	0.7940	0.696–0.872	63.33%	83.33%
	SM(d18:1/18:1(11Z))				
C	PC(o-16:1(9Z)/18:0)	0.8960	0.813–0.950	90.00%	80.00%
	PC(20:4(8Z,11Z,14Z,17Z)/18:0)				
	PC(18:0/22:5(4Z,7Z,10Z,13Z,16Z))				
	SM(d18:1/20:0)				
D	PC(o-16:1(9Z)/18:0)	0.8890	0.805–0.945	80.00%	83.33%
	PC(20:4(8Z,11Z,14Z,17Z)/18:0)				
	PC(18:0/22:5(4Z,7Z,10Z,13Z,16Z))				
	SM(d18:1/18:1(11Z))				
E	PC(o-16:1(9Z)/18:0)	0.8890	0.805–0.945	80.00%	86.67%
	PC(20:4(8Z,11Z,14Z,17Z)/18:0)				
	PC(18:0/22:5(4Z,7Z,10Z,13Z,16Z))				
	SM(d18:1/20:0)				
	SM(d18:1/18:1(11Z))				

## Discussion

Tuberculosis is caused by *Mycobacterium tuberculosis*, one of the most widespread infectious diseases worldwide (Procop, 2016), which is always divided into two types: pulmonary tuberculosis (PTB) and extrapulmonary tuberculosis (EPTB). Because of its lower incidence rate, EPTB does not attract enough attention compared to PTB. Osteoarticular tuberculosis is one of EPTB, whose *M.tb* directly infects the bone and joint tissue or spreads to the bone and joint tissue from other parts, and it is hard to distinguish with rheumatic immune arthritis and bone tumor at its early stage based on current clinical laboratory and clinical imaging methods (Fan et al., 2018). Metabolomics is one of the omics proposed together with proteomics and transcriptomics in recent years (Dutta et al., 2020). It is defined as the study of the complete set of metabolites inside cells, tissues, organs, and biological fluids. It represents a major and rapidly evolving component of systems biology—a new integrative approach to deciphering the complexity of biological systems (Preez et al., 2017). The metabolite changes associated with the specific phenotype being investigated may be classified as characteristics of the perturbation, which, in the context of investigating a disease, could be used toward better disease characterization, diagnostics, treatment, and other clinical applications. The methods of metabolomics include nuclear magnetic resonance (NMR) and mass spectrometry (MS) (Laíns et al., 2019). NMR is appropriate for the detection of all hydrogen-containing compounds, since it determines the magnetic resonance of nuclei in a molecule, and it is considered an unbiased, robust, reproducible, non-destructive, and selective analytical platform, which requires almost no sample pretreatment. On the other side, however, NMR has a low sensitivity and is short of an analyte separation element (Shin et al., 2011). MS is defined as the process of forming gaseous ions, with or without fragmentation, which are then characterized by their *m/z* ratios and respective relative abundances. Direct MS infusion is a high-throughput method, requiring short time for each sample analysis, and has been applied successfully in metabolomics studies, but it is not preferred for the analyses of complex biological samples such as blood and urine due to matrix interference (Schoeman et al., 2012). For TB, there are also more metabolomics studies focusing on colony culture, sputum specimens, blood specimens, urine specimens, tissue specimens, etc. (Parida and Kaufmann, 2010)

Zhou et al. (2015) and Albors-Vaquer et al. (2020) used NMR to detect differences in the expression of metabolites in the serum of PTB, healthy adults, lung-related benign lesions, and lung cancers; the results showed that some amino acids have been changed among these groups, such as alanine, lysine, glutamate, glutamine, ketone bodies, lactate, and pyruvate. Mendes Rˆego et al. (Rêgo et al., 2021) used MS to detect serum metabolites among drug-sensitive tuberculosis and drug-resistant tuberculosis; there were also some amino acids that were changed such as isoleucine, proline, hercynite, betaine, and pantothenic acid. On another aspect, Vrieling et al. (2019), Luo et al. (2020) and Chen et al. (2021) used MS to detect serum metabolites among PTB and healthy control, tuberculous pleuritis and malignancy, and PTB and PTB with type 2 diabetes, respectively. The results of these studies also showed some changes in amino acids, phospholipids, sphingolipids, etc., which are similar to other studies. According to other studies, amino acid levels such as alanine, lysine, glutamate, and glutamine in patients with tuberculosis infection have decreased significantly, which may be related to the uptake of glutamate, glutamate, and alanine by *Mycobacterium tuberculosis* for their corresponding life activities (Harth and Horwitz, 2003; Agapova et al., 2019). The body’s immune process for tuberculosis is mainly the activation of T cells, which also changes the law of glucose metabolism. The disease process affects the metabolism of related immune cells and thus affects the secretion of cytokines such as IFN-γ, further leading to a decrease in immune effect (Lande et al., 2003).

According to the characteristics of *Mycobacterium tuberculosis*, in addition to the cell membrane and peptidoglycan of ordinary bacteria, there are also a large number of lipids and carbohydrates on its surface. These components have strong biological activity on eukaryotic cells, thus suggesting that it has a strong relationship with pathogenicity. For the mechanism of tuberculosis, lipid metabolism and lipid effector molecules play a vital role, such as regulating the production of cytokines, scavenging oxygen free radicals, and producing granulation inflammation and mitochondrial toxicity (Walpole et al., 2018). These related pathogenic components have also been reflected in other studies. Our study focuses on the blood specimens of osteoarticular tuberculosis, compared with PTB; this type of research is currently rarely reported. The results of our study showed that compared with healthy adults and rheumatoid arthritis patients, patients with osteoarticular tuberculosis also have significant changes in serum amino acid and lipid metabolism. Most amino acids such as glycine, L-arginine, and L-alanine were downregulated in osteoarticular tuberculosis patients’ serum; in contrast, most lipids such as phosphatidylcholine, sphingomyelin, and phosphatidylethanolamine were upregulated in osteoarticular tuberculosis patients’ serum.

Further, our study selected five potential serum metabolite biomarkers, namely, PC(o-16:1(9Z)/18:0), PC(20:4(8Z,11Z,14Z,17Z)/18:0), PC(18:0/22:5(4Z,7Z,10Z,13Z,16Z)), SM(d18:1/20:0), and SM(d18:1/18:1(11Z)), which belong to phosphatidylcholine (PC) and sphingomyelin (SM). On the aspect of KEGG enrichments, necroptosis, choline metabolism, sphingolipid signaling, retrograde endocannabinoid signaling, sphingolipid metabolism, and glycerophospholipid metabolism are maybe the main pathways. In most cases, these metabolites are related to lipid accumulation and obesity in the body (Wahl et al., 2012; Reinehr et al., 2015; Hellmuth et al., 2016). At the same time, some other pathological processes that cause enhanced lipid metabolism can also significantly increase these metabolites; for example, Wu et al. (2018) have reported that the sphingomyelinase/ceramide system, which has shown several times to be a crucial factor in the internalization, processing, and killing of diverse pathogens, also modulates the pro-inflammatory response and the state of mycobacteria in macrophages, which highlights the important role of lipid metabolism in the pathogenic mechanism of pathogens. For PTB, sphingosine-1 phosphate (S1P) and ceramide are central molecules and are decisive for sphingolipid signaling; otherwise, they are about the secretion of interferon (IFN)-γ during the course of infection and infiltration of pulmonary CD11b+ macrophages and expression of S-1P receptor-3 (S-1PR3) in the lungs during the course of infection (Braverman et al., 2016; Nadella et al., 2019). Takenami et al. (2018) have a research about the IgM and total IgG antibody response to cardiolipin (CL), phosphatidylcholine (PTC), phosphatidylethanolamine (PE), phosphatidylinositol (PI), and sulfatide (SL-I) as biosignatures that can be used to diagnose PTB and its applicability for monitoring the efficacy of antituberculosis treatment; the antibody concentrations of PTB patients were significantly higher than those of healthy control, which also indicates that lipids play a vital role in the process of *Mycobacterium tuberculosis* infection. Also, fatty acids can stimulate the activation of dormant *Mycobacterium tuberculosis* in liquid medium (Shleeva et al., 2013). Eicosanoids, lipid mediators derived from arachidonic acid, have been associated with the modulation of the host response to *Mycobacterium tuberculosis* infection (Bafica et al., 2005; Tobin et al., 2012). Moreover, it also reported increased eicosanoid ratios in plasma in tuberculosis patients compared to healthy control (Mayer-Barber and Sher, 2015). However, related research about the lipid in osteoarticular tuberculosis or even other extrapulmonary tuberculosis is rare; the results of our research, whether from the differential metabolites screened out or the signal pathways obtained from bioinformatics, are closely related to the results of pulmonary tuberculosis-related research (Huang et al., 2020; Han et al., 2021). It provides more reference value for future research on diagnostic biomarkers from the perspective of metabolomics and provides some hints for the establishment of subsequent auxiliary diagnostic methods for these differential metabolites.

In the future study, we will expand the scope of clinical serum samples, from the single-center collection to the multicenter collection in different regions, in order to minimize the limitations of the experimental results. At the same time, serum samples of different tuberculosis diseases such as pulmonary tuberculosis can be added as follow-up controls to determine whether the relevant markers are widely related to tuberculosis, and then extended to not only the auxiliary diagnosis for osteoarticular tuberculosis but also the establishment of a new method of laboratory examination for tuberculosis for reference.

## Data Availability Statement

The data presented in the study are deposited in the MetaboLights repository, accession number MTBLS4187 with URL www.ebi.ac.uk/metabolights/MTBLS4187.

## Ethics Statement

The studies involving human participants were reviewed and approved by the Ethics Committee of Chinese PLA General Hospital. The patients/participants provided their written informed consent to participate in this study.

## Author Contributions

CW and HL contributed to the research design. XC performed the experiments, coordinated the data modeling, and wrote the paper. JY performed the collection of serum samples and subjects’ clinical data. All authors contributed to the article and approved the submitted version.

## Conflict of Interest

The authors declare that the research was conducted in the absence of any commercial or financial relationships that could be construed as a potential conflict of interest.

## Publisher’s Note

All claims expressed in this article are solely those of the authors and do not necessarily represent those of their affiliated organizations, or those of the publisher, the editors and the reviewers. Any product that may be evaluated in this article, or claim that may be made by its manufacturer, is not guaranteed or endorsed by the publisher.
