# Supplementary material for: Novel Potential Diagnostic Serum Biomarkers of Metabolomics in Osteoarticular Tuberculosis Patients: A Preliminary Study
Source: Front Cell Infect Microbiol. 2022 Mar 25;12:827528. doi: 10.3389/fcimb.2022.827528 (PMC8992656; doi:10.3389/fcimb.2022.827528)
Supplement: Supplementary file 1 [file DataSheet_1.pdf]

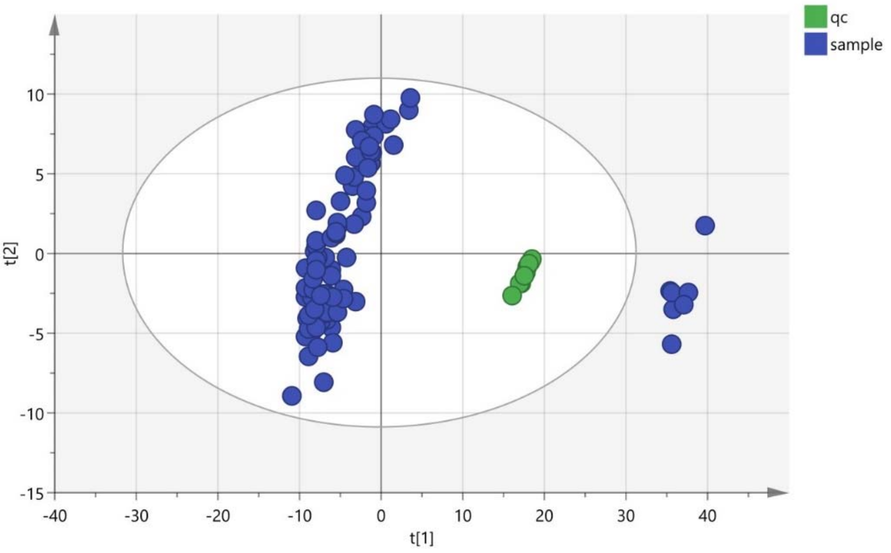

Figure 1 QC result of reversed-phase chromatography positive ion pattern

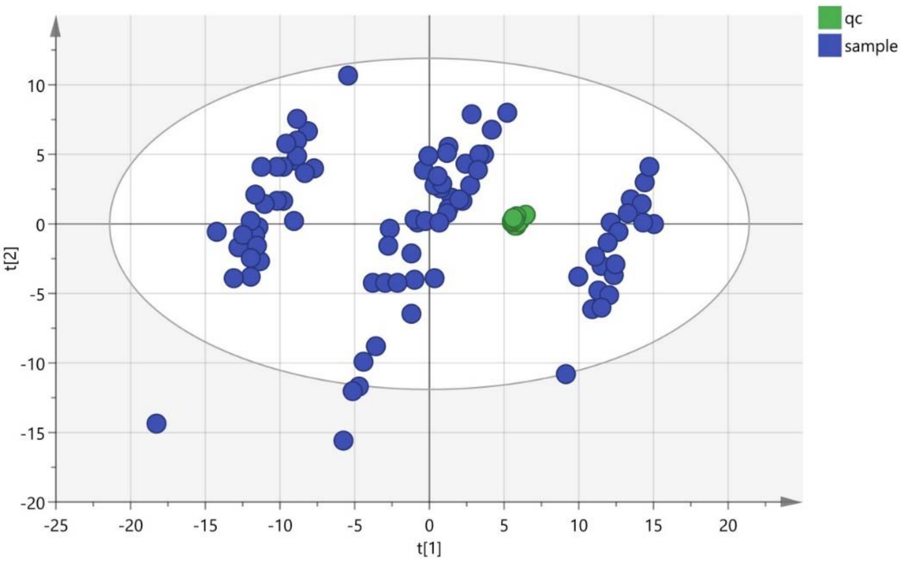

Figure 2 QC result of reversed-phase chromatography anion pattern

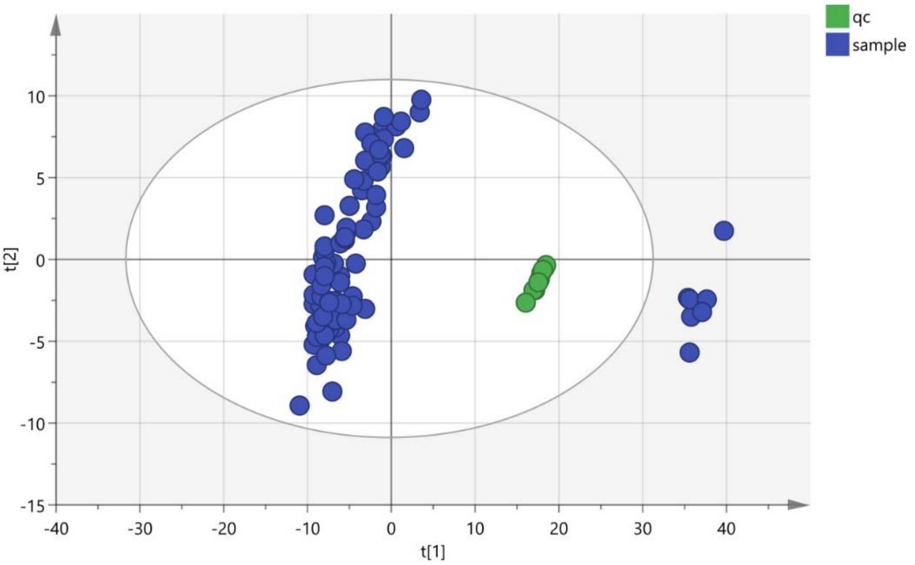

Figure 3 QC result of hydrophilic chromatography positive ion pattern
